# Knowledge and Challenges Associated With Hearing Impairment in Affected Individuals From Cameroon (Sub-Saharan Africa)

**DOI:** 10.3389/fresc.2021.726761

**Published:** 2021-11-18

**Authors:** Edmond Wonkam-Tingang, Karen Kengne Kamga, Samuel Mawuli Adadey, Seraphin Nguefack, Carmen De Kock, Nchangwi Syntia Munung, Ambroise Wonkam

**Affiliations:** ^1^Division of Human Genetics, Faculty of Health Sciences, University of Cape Town, Cape Town, South Africa; ^2^West African Centre for Cell Biology of Infectious Pathogens (WACCBIP), University of Ghana, Accra, Ghana; ^3^Department of Paediatrics, University of Yaounde 1, Yaounde, Cameroon

**Keywords:** hearing impairment, perception, psychosocial challenges, genetic counselling, Cameroon, Africa

## Abstract

**Background:** This study aimed to gain an understanding of the challenges faced by people with hearing impairment (HI) in Cameroon, their understanding of the causes of HI, and how challenges could be remedied to improve the quality of life of persons with HI.

**Methods:** Semi-structured one-on-one in-depth interviews and observation of participant behaviour when answering questions were used to collect data from 10 HI professionals (healthcare workers and educationists), and 10 persons affected by HI (including caregivers).

**Results:** The results show that the different groups associate the causes of HI to genetics, environmental factors, and a spiritual curse. There were reported cases of stigma and discrimination of persons with HI, with people sometimes referring to HI as an “intellectual disorder.” Interviewees also highlighted the difficulty persons with HI have in accessing education and healthcare services and suggested the need for the government and health researchers to develop strategies for the prevention and early diagnosis of HI. These strategies include (1) the awareness of the general population regarding HI, (2) the development of facilities for the proper management and new-born screening of HI, and (3) the implementation of a premarital screening to reduce the burden of HI of genetic origin.

**Conclusions:** This study confirms the difficult social interaction and access to proper management faced by persons with HI in Cameroon and further highlights the need to educate populations on the causes of HI for a better acceptance of individuals with HI in the Cameroonian society.

## Background

Hearing is a major sensation (1) in humans and plays critical roles in human-human and human-environment interactions (2). People with hearing impairment (HI) have partial or total inability to hear sound in one or both ears (1). Globally, an estimated 466 million people are currently living with HI, the majority (over 80%) of which are based in low- and middle-income countries (3). It is also anticipated that by 2050, the global estimates will be over 900 million (4). HI can be caused due to genetic or environmental factors, and in many cases, it is not possible to establish a definite aetiology (5–7). Environmental factors such as meningitis, measles, or ototoxicity are the leading causes of HI in low- and middle-income countries, while their burden is lower in high-income countries (6, 8–10). It is thought that ~half of congenital HI cases are of genetic origin (3). Pathogenic variants in *GJB2* gene constitute the most common cause of non-syndromic HI in European and Asian populations (7), while their prevalence is close to zero in sub-Saharan Africa (SSA) (11). Depending on whether there are additional clinical signs or not, genetic HI cases are classified as syndromic or non-syndromic.

Among parents and family members of individuals with HI, there is limited knowledge of the genetic causes of HI (3). A study from Russia showed that most families, especially families without HI history do not associate the aetiology of HI to genetic/inheritable factors (12). Rather, they often cite causes such as premature birth, trauma at birth, infection, Rhesus incompatibility, drug intoxication, pregnancy complications, maternal rubella, and otitis (12). This limited knowledge of the genetic causes of HI may be more problematic in Africa, where perceived causes of deafness vary from environmental factors to mysterious (“evil forces”) or superstitious beliefs (13, 14). For example, a study in South Africa reported that few parents associated their child's HI with genetic causes while most explained it to recurrent infections (3). In the South African study, parents who reported genetic causes were likely to have been in contact with the genetics unit at their hospital as part of their child's healthcare (3). In a review on HI in SSA, Kiyaga et al. (15) stated that HI is often associated with mysterious fate, and, in some cases, “God's will.” Similarly, studies in Nigeria and South Africa revealed that the majority of traditional healers ascribed HI to supernatural causes (14, 16) and other superstitious beliefs (13).

In most cases, children with HI are born from hearing parents who have little or no experience with the HI (17). Thus, parents of children with HI tend to face challenges of parenting, some of which may affect the family structure especially in terms of communication and social interaction (17). Access to basic social services for children with HI and helping them interact with extended family members are additional challenges faced by parents (17). It is therefore essential to help parents and/or caregivers of children with HI to navigate some of these psychosocial challenges, especially in terms of sign language communication and interactions with children with HI, and adjusting to the new family dynamics brought about by HI (18, 19).

Previous studies from Cameroon (a sub-Saharan African lower middle-income country) assessed the challenges faced by persons with HI, and found that the attitude of the society towards individuals with HI does not encourage their participation and involvement in the community, as they are often discriminated against (20). Additionally, persons with HI were shown to have limited access to education in Cameroon, as they have few opportunities to further their education (21). The understanding of persons with HI of the causes of their condition, and their expectations towards policymakers in Cameroon, however, remain elusive. In the present study, we sought to revisit the challenges faced by persons with HI in Cameroon, explore their understanding of the causes of HI, and how challenges can be remedied to improve the quality of life of persons with HI.

## Methods

### Study Sites and Population

Individuals selected for inclusion in this study were residing in two rural (Far-North, and North) and one urban (Centre) regions of Cameroon. The latter has a population of ~24,053,727 (22), and a HI prevalence ranging from 0.9 to 3.6% (7). Of these, environmental factors such as meningitis, impacted wax, and age-related disorders contribute to more than half of all recorded HI cases, ~14.8% is due to hereditary causes while in the remainder of cases, the cause is unknown (7).

This qualitative study is part of a bigger project (HI-GENES Africa) that aims to study the genetic aetiology of HI in Cameroon. The three study sites were the starting points for the broader study. These regions have well-established schools and institutions for the deaf, thus presenting a good opportunity to recruit HI professionals, persons with HI, and their parents/caregivers. For example, of the 19 special schools for individuals with HI in Cameroon, nine are located in the study sites (three in the Far-North, three in the North, and three in the Centre regions) and are all privately run. In these well-established schools, the communication method is sign language, and most teachers are qualified sign language interpreters. Hearing health care in Cameroon is mainly supported by the private sector. Indeed, hearing tests (including audiometry and tympanometry) and devices (when available) are mainly provided by private schools and institutions for the deaf, with limited availability in some public hospitals. Also, all the centres that provide speech therapy in Cameroon are privately run.

We included individuals that were either affected by HI (i.e., persons living with HI, and their family members), or implicated in the management of people living with HI (i.e., professionals working in the field of HI), and who freely consented to participate in the study. Participants were divided into two groups: (1) professionals who provide health or educational services specific to HI and (2) families with a child with HI. The group of professionals (*n* = 10) was made of three directors of schools, three specialised teachers, one audiologist, one audiology technician, one speech therapist, and one ear nose and throat (ENT) specialist. The group of families (*n* = 10) consisted of three persons with HI, four parents, and three siblings. We distinguished two types of families: (1) multiplex families (*n* = 6; including three parents and three siblings) where at least two individuals in the same sibship have HI, and the disease was of genetic origin; and (2) singleplex families (*n* = 4; including three persons with HI and one), where only one individual with HI was found, and an environmental factor was identified as the cause of the disease. Participants were selected through schools for the deaf, and family members via community engagement activities.

### Interview Guide and Data Collection

We conducted an exploratory survey, and data were collected through one-on-one semi-structured interviews and observation of participant behaviour when answering questions (i.e., laughing, crying, etc.) (23). In reporting the results, participants' privacy, and confidentiality, was protected through the use of pseudonyms.

A total of 20 interviews were conducted in a location convenient for the interviewee and in their preferred language (17 in French, and 3 in French sign language). A qualified sign language interpreter certified by the Ministry of Higher Education of Cameroon assisted with the interviews (*n* = 3) that involved persons with HI, as the latter communicated through sign language only. A semi-structured interview guide was used to explore perceptions of HI including causes of HI, the psychosocial burden associated with HI based on their lived experiences and means to overcome challenges. Before the interviews, participants completed a short questionnaire to collect socio-demographic information. All the interviews were digitally audio-recorded with consent, including those conducted with help from the sign language interpreter (the interpreter was recorded and considered as reflecting the participant's responses).

### Data Analysis

Audio-recordings were transcribed verbatim and translated from French to English. To familiarise ourselves with the data, each transcript was read several times. The transcripts were imported into the qualitative data analysis software NVivo 12 Pro, and each of them was subjected to content analysis (24). We used the thematic analysis method to identify themes within the data (25). Thematic analysis implies identifying, analysing, and reporting patterns (themes) within data (25).

## Results

### General Characteristics of the Participants

A total of 20 participants were interviewed, including 12 males and 8 females, with ages ranging from 16 to 54 years. Most of participants (13/20) lived in a rural area, while a few (7/20) were residing in urban settings. At the time of the interviews, most participants had a formal secondary education level (14/20), and the remaining six had obtained a tertiary degree (Table 1).

**Table 1:** Characteristics of participants.

**Category**		
*n*	20	
Mean age (SD)	34.15 (11.36)	
Category of participant	*n*	%
**Gender**		
Male	12	60
Female	8	40
**Home language**		
French	17	85
FSL	3	15
**Marital status**		
Single	10	50
Married	8	40
Divorced	1	5
Widow	1	5
**Area of residence**		
Rural	13	65
Urban	7	35
**Level of education**		
Secondary	14	70
Higher	6	30
**Relationship with HI**		
Professionals	10	50
Families	10	50

*FSL, French sign language; HI, hearing impairment; SD, standard deviation*.

**Figure 1:**
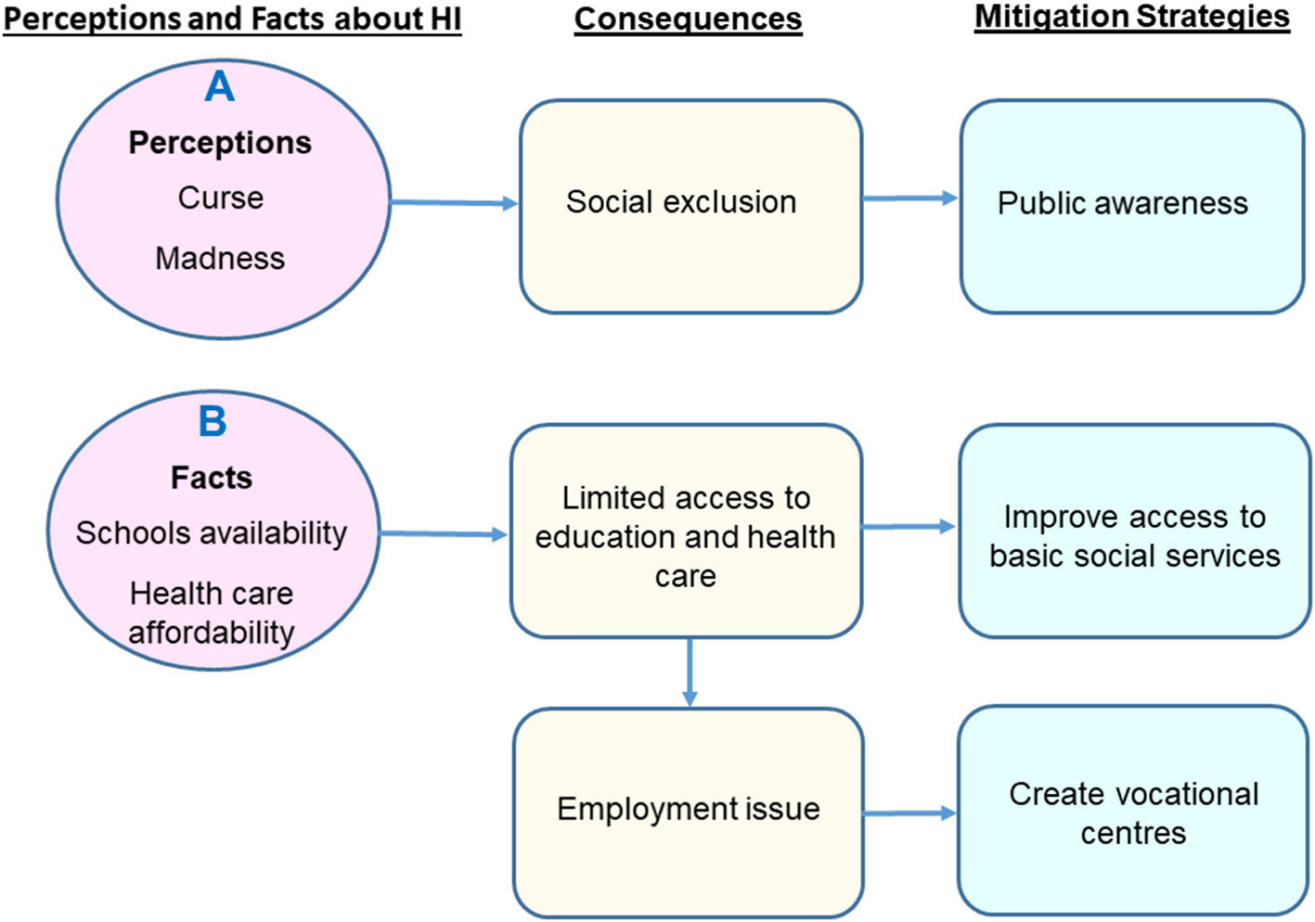
Summary of the main findings. **(A)** The misunderstanding of the causes of HI leads to the social exclusion of people with HI. **(B)** The limited access to education and health care services constitutes a barrier to education and proper management.

The results will be reported based on three themes: (1) perception of HI; (2) challenges faced by the HI community in Cameroon; and (3) expectations of the HI community to policymakers and researchers (Figure 1, Table 2).

**Table 2:** Summary of themes and subthemes with selected quotes.

**Themes**	**Subthemes**	**Selected quotes**
Perception of HI	Hearing disability	“*Basically, it is a disease that manifests through the inability of a person to hear or talk” (Participant 008, brother of a person with HI)*
		“*Hearing impairment means that there is a dysfunction of the ear. Hearing impairment can be total, moderate, severe, or profound.” (Participant 004, female, specialised teacher)*.
	Curse	“*Well, some of them think that deafness is a curse, you have been cursed, the person has been cursed. Some people think like that. And if there are several cases of deafness in a family, they will say that it is a curse” (Participant 008, brother of a person with HI)*.
		“*For them, it is like witchcraft, especially because I am a stranger, I am in an x (pseudonym) family. On my side, my family is saying x (pseudonym) put witchcraft on children. On my husband's side, they say that the witchcraft from my family has affected the children. It is very difficult.”(Participant 019, mother of three children with HI)*.
	Inherited disease	“*According to me, it is genetic. Because in my case, for example, the elder brother of my husband is deaf. So, I think that these genes are in my husband. That is why my children took these genes.” (Participant 019, mother of three children with HI)*.
		“*We can also have congenital hearing impairment cases, which can be inherited. We often have some cases where we have a family with three deaf children while parents are not deaf. Since we know the gene for deafness is recessive, when it is present in both parents, then there is a risk for them to have deaf children.” (Participant 015, male, audiologist)*.
	Acquired disease	“*(sign language translation) When we are seriously sick, we can become deaf. There is the sun, there is meningitis.” (Participant 012, male, student, individual with HI)*.
		“*Hearing impairment has many aetiologies. We have prenatal aetiologies, which are diseases that the mother can suffer from. We also have mismanagement of some diseases, by taking some antibiotics like paracetamol in excess, quinine; that is one of the prenatal aetiologies. We also have perinatal aetiologies. During delivery, the baby can have some trauma, such as anoxia so, the umbilical cord can surround the neck. And the third aetiology is postnatal causes. Here we have shocks. Here, the patient has already acquired speech. Diseases such as meningitis, or noise when wearing speakers on ears can alter hearing.” (Participant 001, male, audiology technician)*.
	Intellectual disability	“*As soon as they realise that a person is hearing impaired, they start treating him like a intellectually disabled person. That is what we observe. They will say that it is madness (laughing). Yes, most of them will say that it is a disease that affects the brain, yes most of them” (Participant 020, male, speech therapist)*.
		“*Most of the time they are called “foolish people”, it means people who are not intelligent, people who are a little crazy, people who react quickly, people who are too stupid. But this is not true.” (Participant 002, male, director of a school for the deaf)*.
Challenges associated with HI	Socialisation	“*When you go to a remote area, they will say this is a “moumou”, since he is here, he is alone in his corner. But this is because people do not understand him.” (Participant 015, male, audiologist)*.
		“*(sign language translation) There is somehow discrimination compared to people who can hear. They are not invited to some meetings; there are side-lined. They think that deaf people are not equal to normal people; they are always side-lined from some meetings. They say that he is deaf, what can he do? What can he say?” (Participant 012, male, student, Individual with HI)*.
		“*People who do not know deafness will reject them. Because they think that they are crazy, they are witches, they do not have all their senses, and they are not considered. But people who know deafness well accept them.” (Participant 003, male, specialised teacher)*.
	Access to basic social services	“*Almost all specialised institutions are private and are therefore sometimes expensive. Parents have to pay tuition fees. Moreover, in addition to that, it is not enough; the government has to allocate funds, for the institution to work efficiently. So we really need funding to help everybody, because they are not a lot, but their management is expensive.” (Participant 005, male, specialised teacher)*.
		“*It is very expensive. First, diagnosing hearing impairment is a problem, especially in children. Apart from the eardrum assessment that we can do everywhere, the Auditory Brain Respond, a specialised exam, is not available everywhere, and it is costly. Moreover, even when the Auditory Brain Respond is done, we have to either operate the child to put a cochlear implant, and we cannot do it in Cameroon. A team has already started doing it, but it is a European team, and it is not common. Secondly, we can prescribe a hearing aid, which is expensive. One implant cost at least five hundred thousand francs that is the salary of four months for a civil servant. This is not affordable for a common citizen.” (Participant 011, male, ENT specialist)*
	Employment	“*Because deaf people cannot hear, I do not see which area we can employ them. Because every working environment requests communication. I do not think that we can employ them in any area” (Participant 008, brother of a person with HI)*.
		“*And when there is a deaf lady in the community, people tend to exploit her for work and so on. So, exploit her in the sense that they don't want to compensate her for the work she did, they will say that she is deaf and she should work.” (Participant 002, male, Director of a school for the deaf)*.
	Autonomy	“*She is a big girl, but she can't travel alone, she can't do anything alone, nothing…. it is difficult. You should always be with her, be her guardian angel, so I can't die? (laughing)” (Participant 016, mother of two children with HI)*.
Expectations	Improve access to basic social services	“*First of all, reduce our tuition fee. Secondly, they should build a high school for the deaf. Because I think there is no high school for the deaf. When they finish primary school, they have to attend a high school for hearing-people; I do not even know how they make it. So, we have to build their high school, and if possible, their university.” (Participant 018, mother of a child with HI)*.
		“*I would like their management to be funded just like the management of other diseases. They should consider deafness as a disease like AIDS, which we fight against, like cancer. So they should act in such a way that even poor parents can afford the treatment, so exams, hearing aids.” (Participant 019, hairdresser, mother of three children with HI)*.
	Public awareness	“*The government should also raise awareness in the society so that when people will see individuals with hearing impairment, they will know exactly who they are; they will not stigmatise them, they will now know their psychology, they will understand that they react like that because of this or that situation. So the society will be informed, and they will have fewer problems.” (Participant 002, male, Director of a school for the deaf)*.
		“*Now your work will help. It will help in the sense that when you publish, people will read it, and they will realise that there is an issue that is neglected. Furthermore, it will help people to understand better, as we said earlier, many people do not even know that it is a problem and that it is not madness (laughing). When I was younger, when I used to see people with hearing impairment, I did not use to consider them, I was asking: what can he do? I think people behave like that just because of their ignorance.” (Participant 020, male, speech therapist)*.
	Reduce the burden of HI of genetic origin	“*I hope that genetics can prevent the next children go through what our children are suffering from now. So I would like doctors to see whether they can help us so that our children do not give birth to deaf children in the future. So if during your research, you can act in such a way that our children do not give birth to deaf children. Just like with HIV, where the mother takes medicine for her baby not to be affected. That will be something great.” (Participant 019, hairdresser, mother of three children with HI)*.
		“*We want doctors to inform people of hearing impairment of genetic origin. You should reach a point where every child who is born is brought to the ENT specialist, just like he is brought to the Paediatrician, before leaving the hospital, in order to diagnose early-onset hearing impairment. Moreover, why not at the level of the laws, we should reach a point that we recommend people to do premarital screening before getting married to not find themselves with children with hearing impairment anymore. So you should make publications, for people to be aware.” (Participant 015, male, audiologist)*.
	Vocational centres	“*The government should take care of these disabled children. They should build a project for them. We have trading projects, a deaf person can easily do trading. If it is not the case, the government can create training centres for them to learn artwork, carpentry, or even dressmaking.” (Participant 001, male, audiology technician)*.

### Perception of HI

Participants mentioned different factors as possible causes of HI. These factors were genetics, an ancestral or religious curse, environmental, or of unknown origins. The perception of the causes of HI in the group of families was influenced by how they are related to HI. Participants from multiplex families tend to associate the occurrence of HI to genetic factors, while participants from singleplex families mainly listed environmental aetiologies.

#### HI as a Hearing Disability

Participants in the group of families defined HI as a disability and referred to it as a disability that is linked not only to loss of hearing but also speech impairment (see quote in Table 2). Participants in the group of professionals defined HI as a decrease in the hearing ability, and further specified that depending on the degree of impairment, it can be classified as mild, moderate, severe, profound, or total (see quote in Table 2).

#### HI as a Cultural or Religious Curse

Most of the participants mentioned that some people in their communities associated HI with witchcraft or a curse (ancestral or religious). This was especially so when a family had many persons with HI, in which case it was considered punishment or curse from a supreme being (i.e., God) (see quote in Table 2). A few interviewees reported that this misconception of HI was also common within their own families, and has, in some cases, led to family disputes and division (see quote in Table 2).

#### HI as an Inherited Disease

Participants from multiplex families mentioned genetics as a primary cause of HI and explained that when parents or grandparents have HI, children can inherit the condition (see quote in Table 2). Interviews with the professionals' group also stated that HI can have a genetic origin and when that is the case it is common to find several affected individuals in the same family. A few of them further stated that the gene that causes HI is recessive (see quote in Table 2).

#### HI as an Acquired Disease

Participants from singleplex families frequently referred to environmental factors such as diseases, climatic conditions, and excessive noise as the main cause of HI. In the interviews, this group cited malaria, neonatal asphyxia, severe flu, headaches, exposition to the sun, or noise as possible aetiologies of HI. The most frequently cited disease was meningitis (see quote in Table 2). In addition to these factors, persons in the professionals' group added that otitis, tumours, the use of ototoxic drugs, and infections such as mumps, measles, and rubella were also responsible for HI (see quote in Table 2).

#### HI as an Intellectual Disability

A few participants from the group of professionals reported that some people in their communities conceptualised HI as a form of intellectual disability or madness. Therefore, they tend to consider people with HI as foolish or not intelligent, and always nervous (see quotes in Table 2).

### Challenges Faced by the HI Community in Cameroon

In the interviews, we also asked participants about the challenges experienced by persons with HI. Participants mentioned several challenges that could be grouped into four areas: socialisation, management, employment, and autonomy issues.

#### Challenges With Socialisation

The participants narrated how individuals with HI are often marginalised and discriminated against both by their own families and society at large, leading to their social withdrawal from their society. This is illustrated by the use of stigmatising labels like “*moumou.”* In Cameroon, the phrase “*moumou”* is often used to refer to persons who use hands to communicate, and what they say or express is construed by the community as mainly due to ignorance or stupidity. This discrimination issue was mostly reported by participants residing in rural areas. Some interviewees explained that children with HI are not respected at home, are treated differently as compared to hearing children, and are most of the time asked to do the most difficult duties (see quote in Table 2).

Participants also explained that people with HI are systematically excluded from community activities. Some interviewees additionally stated how in communities where HI is associated with witchcraft, curse, or intellectual disability, HI individuals experience heightened social exclusion (Figure 1, and quote in Table 2). Overall, HI professionals emphasised the fact that the age of onset and the degree of HI impact on social integration of persons with HI in their communities. For example, a child who did not benefit from early management will have more communication and interaction issues compared to those who had early and appropriate management.

#### Challenges in Accessing Basic Social Services

Most participants highlighted that it is challenging for people with HI to have access to education and healthcare. This was mainly because of their need for sign language translation which is not available in most public and private services. Also, there are very few special schools for persons with HI and most of these schools are privately run not affordable. Some interviewees suggested that this challenge could be overcome through the provision of government subsidies to private institutions to reduce the cost for parents (Figure 1, and quote in Table 2).

In terms of access to health care services, the main challenge was that audiology services are very expensive in Cameroon and many persons are unable to afford the cost. Therefore, even when parents suspect that their child may be having challenges with hearing, they are still unable to seek care in a timeous and consistent manner (see quote in Table 2).

#### Challenges With Gaining Employment

Some interviewees reported that persons with HI have difficulties accessing gainful employment. This is primarily because of their inability to communicate easily with hearing-individuals thereby making job interviews particularly difficult, especially when sign language interpretation is not available as is the case in many institutions in Cameroon. Secondly, even operating within the workplace was a major challenge as they will find it difficult to communicate with colleagues (see quote in Table 2). Furthermore, interviewees estimate that society tends to exploit individuals with HI, as they are sometimes asked to do difficult jobs without any compensation in return (see quote in Table 2).

#### Lack of Autonomy

Participants who had a family member with HI expressed the heavy reliance of the person on their family members for social and financial support. They explained that children with HI are often not able to travel alone or go to school unaccompanied, like other children. This according to the participants adds to the day-to-day workload of the parents. Children with HI are very dependent on their caregivers, which can have an impact on the morale of the caregiver as expressed by one mother of two children with HI who said the dependency of her children is so high that it seems she does not have a right to die (see quote in Table 2).

### Expectations of the HI Community

We also asked participants how the challenges they mentioned could be remedied. They highlighted the need to create more specialised schools (from basic to tertiary) and vocational centres for persons with HI; perform public awareness activities on HI and the challenges; reduce the burden of HI of genetic origin and implement a new-born screening program for HI.

#### Improving Access to Basic Social Services

The major expectation as emerged from the interviews relates to improved access and low-cost education and healthcare. In terms of access to education, they expected the government to fund tuition and training fees of individuals with HI, provide school supplies to existing schools, and create primary schools, high schools, and even universities for people with HI (see quote in Table 2).

In terms of improving access to healthcare, some participants suggested the need for a comprehensive public health or national control program for HI, as is the case with HIV/AIDS, Malaria, and Tuberculosis. If that is done, they hope it will improve the affordability of audiology services (see quote in Table 2). The professionals expressed a need for more healthcare workers who are specialised in the management of HI as well as improved infrastructure for their care.

#### Create Awareness on the Causes of HI and Challenges Experienced by Persons With HI

Participants highlighted the need for the government to raise awareness amongst the general population about HI, including the need not to discriminate against them in terms of access to education, employment, and basic social services. For some participants, public awareness could mitigate negative religious and cultural considerations surrounding HI, which will reduce the stigmatisation and marginalisation currently experienced by the HI community (see quote in Table 2). Some participants mentioned how research programs could also be used as a platform for raising public awareness of HI (see quote in Table 2).

#### Reduce the Burden of HI of Genetic Origin and Implement New-Born Screening for HI

In addition to using research programs to raise public awareness of HI, interviewees from multiplex families expect researchers in the field of genetics to develop strategies to prevent future generations from having children with HI. Using HIV as an example, one participant narrated how with advances in HIV research it is now possible for a pregnant woman to take some medication to prevent the transmission of the infection to her child and suggested that it could be the same for HI (see quote in Table 2).

Professionals expected genetic research to help implement premarital genetic testing to assess the risk of having children with HI. This, they suggested, could also help policymakers to implement legislation that would in the future make premarital genetic testing for HI compulsory. There was also the need for units within the hospital that could support new-born screening for HI (see quote in Table 2).

#### Creation of Vocational Centres

Interviewees, in general, expect the government to train individuals with HI and facilitate their employment. They suggest funding of self-employed projects, favouritism of people with HI as other disabled individuals when it comes to employment, creating training centres for the professional training of people with HI in areas such as sewing of dresses, carpentry, artwork, trading, and any manual works (Figure 1, and quote in Table 2).

## Discussion

This study is, to our knowledge, one of the first reports from Cameroon to provide data on the knowledge, and perceptions of HI, the impact of HI on the daily life of people with HI and their families, and their expectations towards policymakers and researchers. The findings emphasised challenges that are similar to studies reported in other parts of SSA (15, 26).

The data show that the occurrence of HI in Cameroon is sometimes associated with a curse; also, individuals with HI are often considered as being intellectually disabled. This association of HI with supernatural forces has also been described in other African countries (15). In Ethiopia, for example, some communities believe that individuals with HI are possessed by the devil and should be cured by witchcraft or purified water (15), while in Rwanda, some people perceive HI as foolishness (15). This perception of HI is likely due to the non-understanding of the causes of the disease, and suggests thus, the need for more awareness on the causes of HI. Understanding the cause of the disease is critical for the well-being and social integration of the people with HI, as our study found that in communities where HI is associated with supernatural beliefs, individuals with HI experience heightened social exclusion. The social exclusion of people with HI described by the interviewees was previously reported in Cameroon by De Clerck (20), and in Burundi (26). Individuals with HI interviewed in the study by De Clerck (20) reported discrimination against people with HI in that in social events such as family meetings their decision-making ability was sometimes questioned.

The findings of this study showed that persons with HI in Cameroon face challenges in accessing basic social services. Indeed, the few special schools for people with HI in Cameroon are all privately run and are expensive; also, there are currently no high schools for persons with HI in Cameroon. This undoubtedly contributes to the lack of education and ultimately future employment of individuals with HI. The high cost means that for families who are unable to afford tuition, there is the likelihood their child with HI may end up not being able to use sign language and by extension unable to access education and healthcare services even if sign language interpreters are available. The limited number of special schools currently available in Cameroon implies that most families likely stay far from schools, adding thus an additional difficulty in accessing education. The limited access to education observed in the present study has been described in a study conducted previously in Cameroon (21). A study from Canada also reported issues with the education of persons with HI, as teachers indicated that education programs had insufficiently prepared them to teach students with HI effectively (27).

With respect to access to healthcare, there is no universal health insurance scheme in Cameroon, therefore, families pay for every treatment related to HI. Also, the lack of some therapeutic procedures such as cochlear implantation in Cameroon constitutes an additional barrier to the management of people with HI. This difficulty for people with HI to have access to basic services was also been reported in Nigeria (28). To improve the access of individuals with HI to basic social services, we recommend the creation and implementation of a national programme for HI and other disabilities, which will be inspired by existing programmes in the country, e.g., malaria, tuberculosis, and onchocerciasis programs. The functioning of this program as inspired by existing programmes will be based on funding from the government and its international partners. While waiting for the creation and implementation of public schools of the deaf, the government through this national programme will be able to partner with existing private schools and allow children with HI to attend these schools. The newly created national programme will ensure the availability of hearing tests and devices, and assist individuals requiring rehabilitation therapies only currently available in the private sector (e.g., speech therapy).

To reduce the burden of HI of genetic origin in Cameroon, participants suggest the establishment of premarital genetic testing for HI. Coupled with genetic counselling, premarital screening is designed to determine whether individuals carry a genetic predisposition that may produce disease in their offspring, it includes premarital health counselling and a general medical examination, and is particularly important in the prevention of the spread of diseases (29). The successful implementation of a premarital genetic screening programme is thus dependent on the existence of well-established genetic counselling services and also a good understanding of the genetic cause of the disease. However, there is currently to our knowledge no genetic counselling or medical genetics clinics in Cameroon. Also, the genetic aetiology of HI in SSA in general and in Cameroon particularly is not well-known (7). Indeed, Mutations in *GJB2* gene which are the major causes of HI in European, Asian, and Arab populations have a prevalence close to zero in Cameroon and most SSA countries, suggesting that other genes are implicated in HI of genetic origin in Africans (11, 30, 31). It is therefore critical to train genetic counsellors and intensify genetic research on inherited HI, to identify the causative genes and variants, and to complement the genetic counselling of families.

A major limitation of this study is that participants were recruited from only 3 of the 10 administrative regions of Cameroon. Furthermore, because of the limited number of participants, we may not confidently generalise the findings of this study at the national level. There is therefore a need for additional studies with larger sample size to capture the nationwide perceptions and challenges associated with HI in Cameroon. Also, the three individuals included in our study were from well-established schools for the deaf, therefore, they had access to education and some awareness. However, this study explores various aspects of HI, and reports for the first time on the expectations of deaf individuals in Cameroon (including improving access to basic social services, creating awareness on the causes of HI and challenges experienced by persons with HI, reducing the burden of HI of genetic origin and implement new-born screening for HI, and creating vocational centres), and their understanding of the causes of their HI. This study also has a public health impact, as it will aware policymakers of the issues faced by individuals with HI along with suggested mitigations strategies (Figure 1).

## Conclusions

There is a need to educate the general population on the causes of hearing impairment; this should mitigate negative thoughts surrounding HI and allow a better acceptance and integration of people with HI in society. This study shows that the cause of HI can be perceived as a curse like other communities, and people with HI are faced with stigma, discrimination, and difficult access to proper health care management. Additionally, policymakers should invest in creating more special schools, training more teachers and healthcare practitioners, equipping healthcare services, and funding the management of HI to improve the access of persons with HI to basic social services. Lastly, participants were concerned about the occurrence of inherited HI cases and recommended the implementation of premarital screening to reduce its burden. This highlights the necessity to train genetic counsellors and intensify genetic research to identify the genes that contribute to inherited HI cases in Cameroon to provide appropriate genetic counselling to families.

## Data Availability Statement

The raw data supporting the conclusions of this article are available upon request. Queries should be directed to the corresponding author (ambroise.wonkam@uct.ac.za).

## Ethics Statement

Research ethics approval was obtained from the Institutional Research Ethics Committee for Human Health of the Gynaeco-Obstetric and Paediatric Hospital, Yaoundé, Cameroon (No. 723/CIERSH/DM/2018) and the University of Cape Town's Faculty of Health Sciences' Human Research Ethics Committee (HREC 484/2019). All participants provided written informed consent, including permission to digitally audio record interviews and to publish the findings of the study. In the case of minors (<21 years), informed consent was obtained from parents, with the verbal assent of the participant, all these in respect of the Declaration of Helsinki (32). Written informed consent to participate in this study was provided by the participants' legal guardian/next of kin.

## Author Contributions

AW: conceptualisation. EW-T: interviews, transcription and translation of interviews, and coding of the transcripts. KKK, SN, and NSM: crosscheck of transcriptions and translations. KKK and CDK: crosscheck of the coding. EW-T and SMA: issue of the first draft of the manuscript. EW-T and NSM: compilation of the revisions. AW supervision of the whole study. All authors reviewed and edited the manuscript and have agreed to the final version of the paper.

## Funding

This research project was supported by the African Academy of Science/Wellcome Trust, Grant Number H3A/18/001 to AW and the NIH (USA), Grant Number U01-HG-009716 to AW. The funders did not play any role in data collection and analysis, and decision to publish.

## Conflict of Interest

The authors declare that the research was conducted in the absence of any commercial or financial relationships that could be construed as a potential conflict of interest.

## Publisher's Note

All claims expressed in this article are solely those of the authors and do not necessarily represent those of their affiliated organizations, or those of the publisher, the editors and the reviewers. Any product that may be evaluated in this article, or claim that may be made by its manufacturer, is not guaranteed or endorsed by the publisher.

## Abbreviations

ENT: Ear nose and throat
FSL: French sign language
HI: Hearing impairment
HI-GENES: Hearing impairment genetic studies
SD: Standard deviation
SSA: Sub-saharan Africa.
