# Supplementary material for: Knowledge and Challenges Associated With Hearing Impairment in Affected Individuals From Cameroon (Sub-Saharan Africa)
Source: Front Rehabil Sci. 2021 Nov 18;2:726761. doi: 10.3389/fresc.2021.726761 (PMC9397862; doi:10.3389/fresc.2021.726761)
Supplement: Supplementary file 1 [file Data_Sheet_1.PDF]

SUPPLEMENTARY MATERIALS

Knowledge and Challenges Associated with Hearing Impairment in Affected Individuals from Cameroon (sub-Saharan Africa)

Edmond Wonkam-Tingang, Karen Kengne Kamga, Samuel M. Adadey, Seraphin Nguefack, Carmen De Kock, Nchangwi Syntia Munung, and Ambroise Wonkam1,

I- Questionnaire

Demographic data: **Code_______________**

1- Name: ____

2- Age (y.o): ____________________

3- Sex: a) male ▢ ; b) female ▢

4- Marital status: a) single ▢ ; b) married ▢ ; c) divorced ▢ ; d) widow ▢

5- Ethnic group: ______________________________________

6- Region of origin: ______________________________

7- Region of current residence: _________________________

8- First language: a) French ▢ ; b) English ▢

9- Second language: a) French ▢ ; b) English ▢

10- Educational level: a) primary ▢; b) secondary ▢; c) higher ▢; d) none ▢

11- occupation: ____

12- Relationship with deafness: a) none ▢ b) I am deaf ▢ c) parent ▢ d) sibling ▢

e) cousin ▢ f) uncle/aunt ▢

g) other relationship ________________________

13- Religion : a) Christian ▢ b) Muslim ▢ c) animist ▢ d) non-religious ▢

e) other_____________________

II- Interview Guide

Can you introduce yourself and tell us more about how you are related to hearing impairment?

Hearing impairment and Genetics

- What do you think hearing impairment (HI) is?
- What do you think can cause HI?
- What do you think genetics is?
- What do you think an inherited disease is?
- In your opinion, can deafness be an inherited disease? Explain?
- Can genetics help you better understand the causes of HI? Explain?
- Do you think genetics can help improve the management of HI? Explain?

Hearing impairment and daily life

- What do you think are the difficulties that deaf people face daily?
- Can deafness be a barrier to the personal development of the deaf? Explain?
- What do you think is the most difficult thing about being deaf?
- What do you think of the integration of individuals with HI in their families?
- What is your opinion on the integration of individuals with HI in the community?
- What do people in your community think of HI?

Hearing impairment and education/employment

- What do you think of the education and employment of persons with HI? Does HI constitute a barrier?
- What do you think of the intelligence and productivity of persons with HI as compared to hearing individuals?

Hearing impairment and economy

- What is your take on the management of HI in Cameroon?
- Do you think HI treatments are affordable to all patients? Can you elaborate?

Hearing impairment and public authorities

- Are you satisfied with what the government is doing for the deaf community? Explain?
- What do you think the government needs to do to improve the development and integration of individuals with HI?

What do you think is the best approach for involving families in HI studies?

Is there anything else you would like to tell us about HI?
